# *Paenibacillus polymyxa* A26 Sfp-type PPTase inactivation limits bacterial antagonism against *Fusarium graminearum* but not of *F. culmorum* in kernel assay

**DOI:** 10.3389/fpls.2015.00368

**Published:** 2015-05-29

**Authors:** Islam A. Abd El Daim, Per Häggblom, Magnus Karlsson, Elna Stenström, Salme Timmusk

**Affiliations:** ^1^Department of Forest Mycology and Plant Pathology, Swedish University of Agricultural Sciences, UppsalaSweden; ^2^Department of Chemistry, Environment and Feed Hygiene, National Veterinary Institute (SVA), UppsalaSweden

**Keywords:** FHB biocontrol, *Paenibacillus polymyxa*, Sfp-type PPTase, lipopeptide antibiotics, bacterial biofilm

## Abstract

*Fusarium graminearum* and *F. culmorum* are the causing agents of a destructive disease known as *Fusarium* head blight (FHB). FHB is a re-emerging disease in small grain cereals which impairs both the grain yield and the quality. Most serious consequence is the contamination of grain with *Fusarium* mycotoxins that are severe threat to humans and animals. Biological control has been suggested as one of the integrated management strategies to control FHB. *Paenibacillus polymyxa* is considered as a promising biocontrol agent due to its unique antibiotic spectrum. *P. polymyxa* A26 is an efficient antagonistic agent against *Fusarium* spp. In order to optimize strain A26 production, formulation and application strategies traits important for its compatibility need to be revealed. Here we developed a toolbox, comprising of dual culture plate assays and wheat kernel assays, including simultaneous monitoring of FHB causing pathogens, A26, and mycotoxin production. Using this system we show that, besides generally known lipopeptide antibiotic production by *P. polymyxa*, biofilm formation ability may play a crucial role in the case of stain A26 *F. culmorum* antagonism. Application of the system for effective strain selection and maintenance is discussed.

## Introduction

*Fusarium* head blight (FHB) is a destructive disease on cereals that is caused by a group of *Fusarium* species including *Fusarium graminearum* and *F. culmorum* (Nazari et al., 2014). FHB is a major threat to agricultural production due to yield losses, but also constitutes a major safety concern when humans and animals consume *Fusarium*-contaminated products due to the accumulation of several mycotoxins (Yazar and Omurtag, 2008; Xiao et al., 2013). Both *F. culmorum* and *F. graminearum* are soil borne and cause not only FHB, but also *Fusarium* foot and root rot on cereals around the globe especially during wet seasons (Scherm et al., 2013). Several mycotoxins are associated with *Fusarium* sp. infection, including deoxynivalenol (DON) and zearalenone (ZEA; Cowger and Arellano, 2013). Higher levels of both toxins in wheat grains are usually connected to infection with *F. culmorum* or *F. graminearum* (Fredlund et al., 2013; Lindblad et al., 2013).

Crop rotation, improved cultivar resistance, or improved fungicide efficacy and timing has benefited producer in previous years on locations with low FHB pressure. However, under conditions that are highly favorable for infection, use of a single management strategy often fails to control the disease and limit myxotoxin production to acceptable levels. To keep DON to manageable levels, a 75% reduction of FHB index must be obtained when the disease pressure is great (Shah et al., 2013). Given that there has been considerable interest in combining the use of fungicides with environmentally friendly methods, biological control has been suggested as one of the integrated management strategies to control these infections. Various of microbial agents are able to inhibit fungal growth. For instance, Shi et al. (2014) reported that some strains of *Bacillus amyloliquefaciens* antagonized *F. graminearum* growth, which significantly inhibited DON production in wheat seeds. Besides biological control of the fungus, decontamination of mycotoxins using bacteria is the other attractive strategy of biological control for the management of mycotoxins in food and feed (Shetty et al., 2007).

The employment of microbes as biological control agents (BCA) against *F. graminearum* and *F. culmorum* have been reported in several studies under greenhouse conditions (Shi et al., 2014). However, BCAs generally do not perform well enough under uncontrolled conditions in soil and they are not used commercially to control FHB (Walter et al., 2010; Matarese et al., 2012). Our BCAs were isolated from soil in Israel, from a field station called Evolution Canyon (Timmusk et al., 2011, 2014a). Earlier, we demonstrated that isolates from the highly stressed south facing slope (SFS) of Evolution Canyon differ remarkably from isolates derived from the more moderate north facing slope (NFS; Timmusk et al., 2011). SFS isolates were significantly better biofilm producers, phosphorus solubilizers and could produce the enzyme 1-aminocyclopropane-1-carboxylate (ACC deaminase; Timmusk et al., 2011). These isolates were also superior in conferring drought tolerance enhancement to treated plants (Timmusk et al., 2014a). Two *Paenibacillus polymyxa* isolates, one from the SFS (A26) and one from the NFS (E1) were used in the present study (Timmusk et al., 2014a).

*Paenibacillus polymyxa* is a plant growth- promoting bacterium (PGPB), with a broad host plant range and is known for its the ability to produce different lipopeptide antibiotics. The first lipopeptide antibiotic was isolated form a rhizosphere isolate in 1947 and since then many antibiotic compounds active against gram-positive and gram-negative bacteria have been reported (Stansly and Schloesser, 1947). *P. polymyxa* is also known as one of the best rhizobacterial biofilm formers – however, its biofilm formation ability varies in habitat dependent manner (Timmusk et al., 2011). One of the SFS isolates, *P. polymyxa* A26, has been found, in field studies, to be very efficient against *Fusarium* sp. (Timmusk et al., 2014b). The genomic sequence of strain A26 reveals that it harbors several giant gene clusters directing the synthesis of bioactive peptides and polyketides (PKs) by modularly organized megaenzymes, i.e., by non-ribosomal peptide synthetases, (NRPS) and PK synthetases, (PKS). Synthesis of both non-ribosomal peptides (NRP) and PKs is dependent on the presence of a functional Sfp-type phosphopantheinyl transferase (PPTase; Mootz et al., 2001). The virulence of various pathogens is dependent on NRP or PK production and therefore their Sfp-type PPTases may be characterized as gate keepers to pathogenicity. For this reason, Sfp-type PPTases have been suggested as a potential target for antibacterial drug development in the medical industry as well as a means of fighting agricultural pathogens (Pieters et al., 2002; Leblanc et al., 2012; Zheng and Burr, 2013).

To study the mode of action of strain A26, we developed a method for A26 mutagenesis (Kim and Timmusk, 2013) and inactivated the A26 Sfp-type PPTase gene resulting in a deletion mutation that is deficient in the production of NRP and PK origin antibiotics and has also lost its antagonistic activity on plate assays. It was confirmed by LC MS/MS that the well-known antibiotic types, fusaricidins and polymyxins, are not produced by the mutant (Timmusk et al., 2015). The mutant is greatly enhanced in biofilm production but is otherwise identical to A26 in other physiological and metabolic parameters (Timmusk et al., 2015). Hence, utilization of this mutant provides an excellent tool to study the mechanism of strain A26 biocontrol (Timmusk et al., 2015). Moreover, strain A26 is particularly attractive for field application; it is a Gram-positive bacterium able to resist pesticides and form endospores that can survive under various stress conditions in the field and has remarkable ability to produce rich biofilms and biologically active compounds.

In order to efficiently utilize strain A26 against *F. culmorum* and *F. graminearum*, one has to know the likely mechanisms of action of this BCA including genes and bioactive compounds involved in the biocontrol mechanisms. Based on this knowledge, it is possible to construct efficient cultivation, formulation and delivery systems of this BCA as well as evaluate its performance under specific field conditions. Here we developed a toolbox comprising of dual culture plate assays and wheat kernel assays including quantitative PCR (qPCR) monitoring of pathogens and agents and mycotoxins produced. Using this system we show that, besides generally known antibiotic production by *P. polymyxa*, biofilm formation ability may play a crucial role in the case of *F. culmorum* antagonism.

## Materials and Methods

### Microbial Agents and Culture Conditions

The fungal pathogens *F. culmorum* strain 42344/1997 and *F. graminearum* strain A602/1998 were obtained from The National Veterinary Institute, Uppsala, Sweden. Both pathogens were grown on potato dextrose agar (PDA) plates at 22°C. *P. polymyxa* A26 and E1 were isolated from the rhizosphere of wild barley at EC SFS and NFS (Timmusk et al., 2011, 2014a) and grown on tryptic soya agar (TSA) at 29°C. *P. polymyxa* A26 Sfp-type- 4′-phosphopantetheinyl transferase deletion mutant strain (A26Δ*sfp*), lacking the ability to produce both NRPs and PKSs was grown under the same conditions.

### Bioassay of *In Vivo* Antagonism

#### Plate Assay

Inhibitory studies between *P. polymyxa* A26 and E1 and *F. culmorum* and *F. graminearum* were conducted on King’s B plates (Ausubel et al., 1988). The bacterial strains were streaked onto the plates after inoculation with fungal plugs. Plates were incubated at 28°C for 5 days.

#### Assay on Wheat Grains

##### Antagonistic Activity of *P. polymyxa* **A26** and **A26**Δ*sfp* Against *F. culmorum* and *F. graminearum*

The antagonistic effect of *P. polymyxa* A26 and A26Δ*sfp* on *F. culmorum* and *F. graminearum* was studied in an experimental setup using sterile wheat grains. Bacterial strains were grown in tryptic soya broth (TSB) overnight at 28°C with shaking at 180 rpm. Cultures were centrifuged for 10 min at 10000 rpm; pellets were washed using sterile water and re-suspended in sterile water. Bacterial cells were counted on Petri plates using the colony forming unit (CFU) method and adjusted to 1 × 10^7^ cells/ml.

Sterile 150 ml conical flasks containing 20 g sterile wheat grains were inoculated with 15 ml 1 × 10^7^ cells/ml *P. polymyxa* A26 or A26Δ*sfp* overnight cultures. Controls were treated with 15 ml sterile water. Flasks were incubated at room temperature for 8 h, and then inoculated with 1 cm^2^ agar plugs from 2 week old cultures of either *F. graminearum* or *F. culmorum*, and incubated at room temperature. Fungal growth was assessed visually and 1 g samples (≈15 grains) were taken from each flask at four time points; i.e., 0, 5, 10, and 15 days after fungal inoculation, and stored at -20°C.

##### Antagonistic Activity of *P. polymyxa* A26 and A26Δ*sfp* Culture Filtrates Against *F. culmorum*

Bacterial strains were grown in TSB at 28°C on a rotary shaker (180 rpm) for 72 h. Cultures were centrifuged for 10 min at 10000 rpm and supernatants were filter sterilized using 0.22 μm Millipore filters. Wheat grains were prepared as described above. Instead of bacterial solutions, equal aliquots of supernatant were used for wheat grain treatments. Controls were treated with 15 ml sterile water. Experiments were performed three times to confirm reproducibility.

### DNA Extraction

The grain samples were freeze dried and ground into a fine powder using a Precellys 24 homogenizer (Bertin Technologies, France). Samples were lysed by incubating 100 mg powder for 15 min in 350 μl of glucose buffer [50 mmol^-1^ glucose, 25 mmol^-l^ Tris-HCl (pH 8), 10 mmol^-l^ EDTA] with 4 mg ml^-1^ lysozyme (Timmusk et al., 2009a). DNA was extracted using a hexadecyltrimethylammonium bromide-based method (Nygren et al., 2008). Pure bacterial, fungal, and plant DNA was also extracted from liquid cultures, mycelia and grains, respectively, using the same method. DNA quantity and quality was assessed with spectroscopic methods using a Nanodrop 1000 (Thermo Scientific, Wilmington, DE, USA).

### PCR and Quantitative PCR Analyses

The presence and amount of *F. culmorum, F. graminearum*, and *P. polymyxa* DNA was monitored by PCR and qPCR. Primers (**Table 1**) targeting EF1α genes in *F. culmorum* and *F. graminearum* as well as wheat were used for PCR as described earlier (Nicolaisen et al., 2009). The bacterial DNA was quantified in the same sample with specific primers designed using Primer3 software (Rozen and Skaletsky, 2000; **Table 1**) targeting the 16S and Sfp-type PPTase gene in *P. polymyxa*. The PCR reaction volume (25 μl) included Dream Taq polymerase enzyme (Thermo, USA) supplemented with 10 μM primers, 10 mM dNTPs, and DNA as a template. As a positive control, a reaction mixture with pure bacterial DNA was used and fungal DNA was used as a template for the negative control reactions. PCR reactions were performed using the following parameters: initial denaturation for 2 min at 94°C; followed by 30 cycles of 1 min at 95°C, 50 s at 54°C, and 1 min at 72°C; and finally 10 min at 72°C in a thermal Mastercycler (ABI, USA). qPCR assays were carried out in 25 μl consisting of 12.5 μl Maxima SYBR Green master mix (Thermo, USA), 0.5 μM primers (forward and reverse) and 100 ng DNA using the Bio-Rad iCycler iQ5 (Bio-Rad, USA). Standard curves consisting of 10-fold dilutions starting at 100 ng DNA for each assay were prepared using pure DNA extracted from either the pathogens or the bacteria. Fungal and bacterial DNA amounts were expressed as a relative ratio to the amount of plant DNA.

**Table 1:** Primers used for PCR and quantitative PCR (qPCR) analysis.

Target	Primer’s ID	Sequence (5^′^–3^′^)	Reference
*Fusarium graminearum*	FgramB F FgramB R	CCATTCCCTGGGCGCT CCTATTGACAGGTGGTTAGTGACTGG	Nicolaisen et al. (2009)
*F. culmorum*	FculC F FculC R	CACCGTCATTGGTATGTTGTCACT CGGGAGCGTCTGATAGTCG	Nicolaisen et al. (2009)
*Paenibacillus polymyxa*	29Pp F 179Pp R	GAGCGGGGTTGATTAGAAGC CTTTCCTCCTTCTCCCATGC	Timmusk et al. (2009a)
*P. polymyxa*^1^	16sA26 F 16sA26 R	GCATGGGAAAAGGAGGAAAG AGCAGTTACTCTACAAGACGTTC	This study
*P. polymyxa*^2^	Sfpdel F Sfpdel R	GTTGGTCTGCCGGCAATTGA GGTTGTCTGCATCCTCACGCA	This study
Plant EF1α	Hor1 F Hor2 R	TCTCTGGGTTTGAGGGTGAC GGCCCTTGTACCAGTCAAGGT	Nicolaisenet al. (2009)

*^1^Target both *P. polymyxa* A26 and A26Δsfp*.

*^2^Target only *P. polymyxa* A26*.

### Mycotoxins Analysis

The concentrations of DON and ZEA were determined in 1 g freeze dried wheat grains at 5, 10, and 15 days after pathogen infection using a LC-MS/MS (Njobeh et al., 2012; Tevell Åberg et al., 2012). Culture filtrate ZEA was quantified using a commercial ELISA kit for ZEA detection (RIDASCREEN, R-Biopharm AG, Darmstadt, Germany) following the manufacturer’s instructions.

### Statistical Analysis and Data Validation:

Data were subjected to analysis of variance (ANOVA) to determine the significance between the different treatments using Costat (CoHort software, CA). Experiments were repeated at least two times.

## Results

### *Paenibacillus polymyxa* A26 and A26Δ*sfp* Antagonism Against Fungal Pathogens on Agar Plates

The antagonistic ability of *P. polymyxa* A26, E1, and A26Δ*sfp* against *F. culmorum* and *F. graminearum* was assayed on King’s B plates (**Table 2**). Both wild-type strains of *P. polymyxa* significantly inhibited the growth of *F. culmorum* and *F. graminearum* where *P. polymyxa* A26 exhibited higher antagonistic activity than strain E1 (**Table 2**). A26Δ*sfp* lost its ability to antagonize both pathogens (**Table 2**).

**Table 2:** *Paenibacillus polymyxa* inhibition of *F. graminearum* and *F. culmorum* on plate assays.

	Inhibition zone (mm)	
Treatments	*F. graminearum*	*F. culmorum*
*P. polymyxa* A26	17 ± 1^a^	16 ± 2^a^
*P. polymyxa* E1	13 ± 1^b^	11 ± 1^c^
*P. polymyxa* A26Δ*sfp*	0^d^	0^d^
Control	0^d^	0^d^

*The antagonistic ability of Sfp-type PPTase mutant was compared with two wild-type *P. polymyxa* strains on King’s B plates. The results are presented as the diameter of the inhibition zone (mm) of fungal growth. Different letters indicate statistically significant differences (*P* ≤ 0.01). The data represent the mean of four biological replicates*.

### *Paenibacillus polymyxa* A26 and A26Δ*sfp* Antagonism Against Fungal Pathogens on Wheat Grains

**FIGURE 1:**
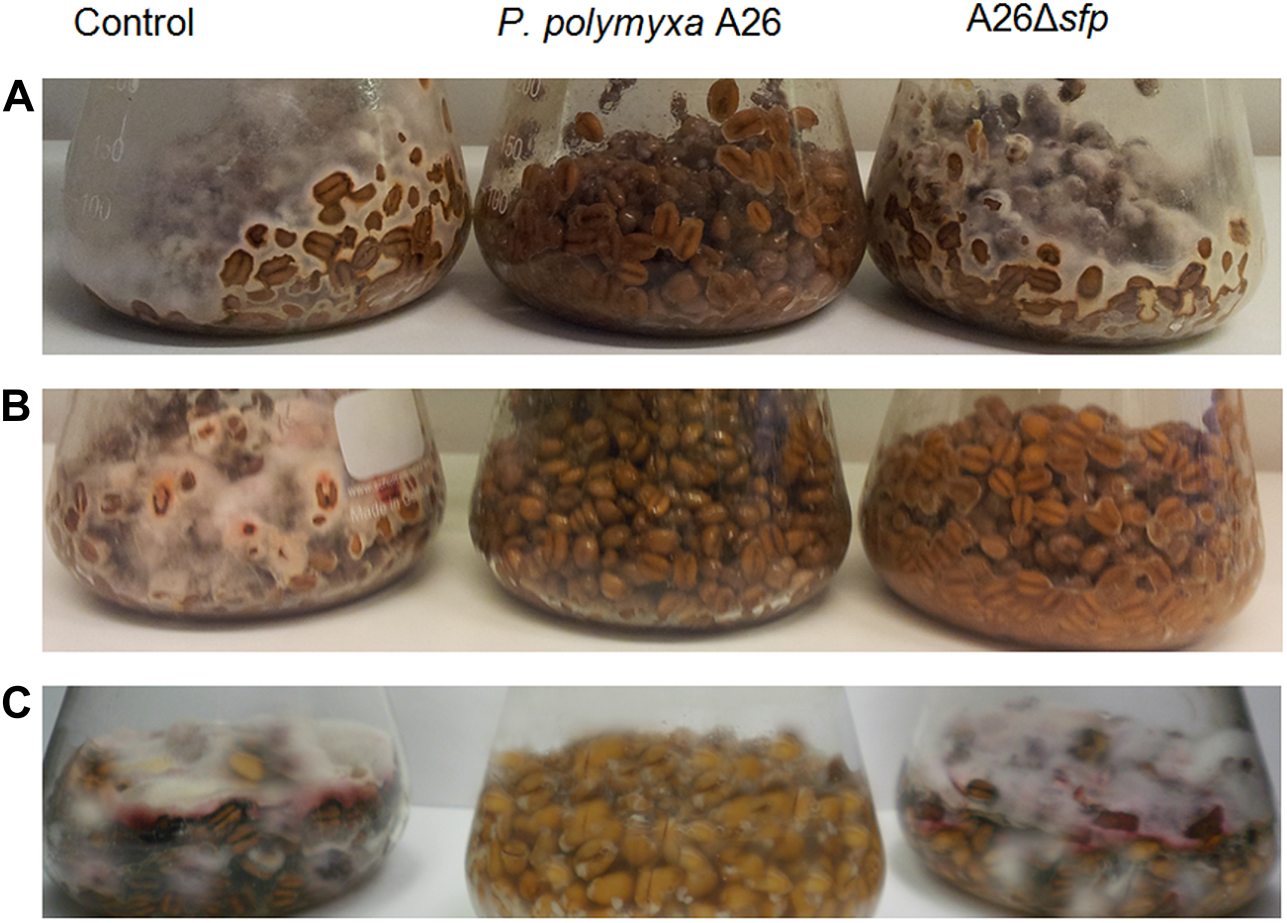
***Fusarium graminearum* and *F. culmorum* antagonism in wheat kernel assay**. *F. graminearum* growth in wheat grains inoculated with *Paenibacillus polymyxa* A26 or A26Δ*sfp* **(A)**, *F. culmorum* inoculated with *P. polymyxa* A26, A26Δ*sfp* **(B)** and *F. culmorum* treated with *P. polymyxa* A26, A26Δ*sfp* culture filtrates **(C)** after 15 days incubation.

Visual inspection of wheat grains over the experimental period revealed increased amounts of *F. culmorum* and *F. graminearum* mycelia in the pathogen control treatment (**Figure 1**). No fungal mycelia was observed on wheat grains treated with *P. polymyxa* A26 (**Figure 1**). The visual observations were followed by pathogen DNA and mycotoxin monitoring. Infection of wheat grains with the pathogens resulted in high DNA and mycotoxin levels (**Tables 3** and **4**). In the absence of bacteria up to 260 and 382 ng pathogen DNA/ng wheat DNA were detected after 15 days for *F. culmorum* and *F. graminearum*, respectively, (**Table 3**). High levels of DON (up to 6.85 mg/kg) were detected in *F. graminearum* samples, while *F. culmorum* strain did not produce any detectable levels of DON (**Table 4**). Accumulation of high levels (up to 61.2 mg/kg) of ZEA were recorded in grains infected with either *F. graminearum* or *F. culmorum* (**Table 4**). No *F. culmorum* and *F. graminearum* DNA nor mycotoxins were detected in *P. polymyxa* A26 treated wheat grains, respectively, (**Tables 3** and **4**). The ability of *P. polymyxa* A26 to inhibit *F. graminearum* growth in wheat grains was significantly compromised by its Sfp-type PPTase inactivation. As shown in **Figure 1**, a considerable growth of *F. graminearum* could be detected in wheat grains treated with A26Δ*sfp* after 15 days. We also detected significant levels of *F. graminearum* DNA (62.66 ng fungal DNA/ng wheat DNA) after 15 days of fungal infection (**Table 3**). Despite very low levels of fungal DNA detected after 5 and 10 days, significant levels of both mycotoxins DON and ZEA were detected at all time points.

**Table 3:** Quantitative PCR (qPCR) analysis for *F. culmorum* and *F. graminearum* DNA (ng fungal DNA/ng wheat DNA).

Treatments	Pathogen DNA (ng Fungal DNA/ng Wheat DNA)		
	5 days	10 days	15 days
*F. culmorum*	206.3 ± 28.9^a^	197.0 ± 62.6^a^	260.7 ± 70.6^a^
A26 + *F. culmorum*	ND^b^	ND^b^	ND^b^
A26Δ*sfp* + *F. culmorum*	ND^b^	ND^b^	ND^b^
*F. graminearum*	42.76 ± 10.9^a^	82.44 ± 24.4^b^	382.38 ± 36.6^c^
A26 + *F. graminearum*	ND^d^	ND^d^	ND^d^
A26Δ*sfp* + *F. graminearum*	0.02^d^	0.03^d^	62.66 ± 17.4^e^

*Fungal DNA was detected at three time points (5, 10, and15 days) after infection. DNA was extracted from samples collected from A26 and A26Δ*sfp* treated as well as untreated wheat grains. Different letters indicate statistically significant differences (*P* ≤ 0.01) within all-time points for each pathogen, based on the LSD test*.

*ND, not detected*.

Unlike what was seen on the plate assays, A26 Sfp-type PPTase mutant antagonized *F. culmorum*. Neither pathogen DNA nor mycotoxins were detected *F. culmorum* DNA in A26Δ*sfp* treated wheat grains (**Figure 1**; **Tables 3** and **4**).

**Table 4:** Deoxynivalenol (DON) and zearalenone (ZEA) contents (mg/kg) in wheat grains inoculated with *F. culmorum* and *F. graminearum*; Both DON and ZEA were detected at three times points (5, 10, and 15 days) after fungal infection.

Treatments	Deoxynivalenol (DON) mg/kg			Zearalenone (ZEA) mg/kg		
	5 days	10 days	15 days	5 days	10 days	15 days
*P. polymyxa* A26 + *F.graminearum*	ND^f^	ND^f^	ND^f^	ND^e^	ND^e^	ND^e^
*P. polymyxa* A26Δ*sfp* + *F. graminearum*	1.5^e^	0.3^e^	0.7^e^	0.24^de^	0.41^d^	0.27^de^
*F. graminearum*	6.9^d^	2.7^b^	1.2^c^	5.93^b^	1.73^c^	10.5^a^
*P. polymyxa* A26 + *F. culmorum*	0	0	0	ND^c^	ND^c^	ND^c^
*P. polymyxa* A26Δ*sfp* + *F. culmorum*	0	0	0	ND^c^	ND^c^	ND^c^
*F. culmorum*	0	0	0	53.7^b^	61.2^a^	55^b^
Culture filtrate treatments						
*P. polymyxa* A26 + *F. culmorum*	0	0	0	ND^b^	ND^b^	ND^b^
*P. polymyxa* A26Δ*sfp* + *F. culmorum*	0	0	0	45.5^a^	54.1^a^	48.0^a^
*F. culmorum*	0	0	0	49.4^a^	52.8^a^	49.7^a^

*The results represent the means of two biological repeats. Different letters indicate statistically significant differences (*P* ≤ 0.01) within all-time points for a single mycotoxin, based on the LSD test*.

*ND, not detected*.

### *Paenibacillus polymyxa* A26 and A26Δ*sfp* Culture Filtrate Antagonism Against *F. culmorum* on Wheat Grains

As A26Δ*sfp* incubation with *F. culmorum* resulted in significant antagonistic ability, further studies were performed to study the effect of culture filtrates from the wild-type and mutant versions of strain A26. Culture filtrates from wild-type A26 efficiently antagonized the pathogen while the A26Δ*sfp* culture filtrated had no significant effect on pathogen growth and mycotoxin production (**Figure 1**; **Table 4**).

### Bacterial Growth

**FIGURE 2:**
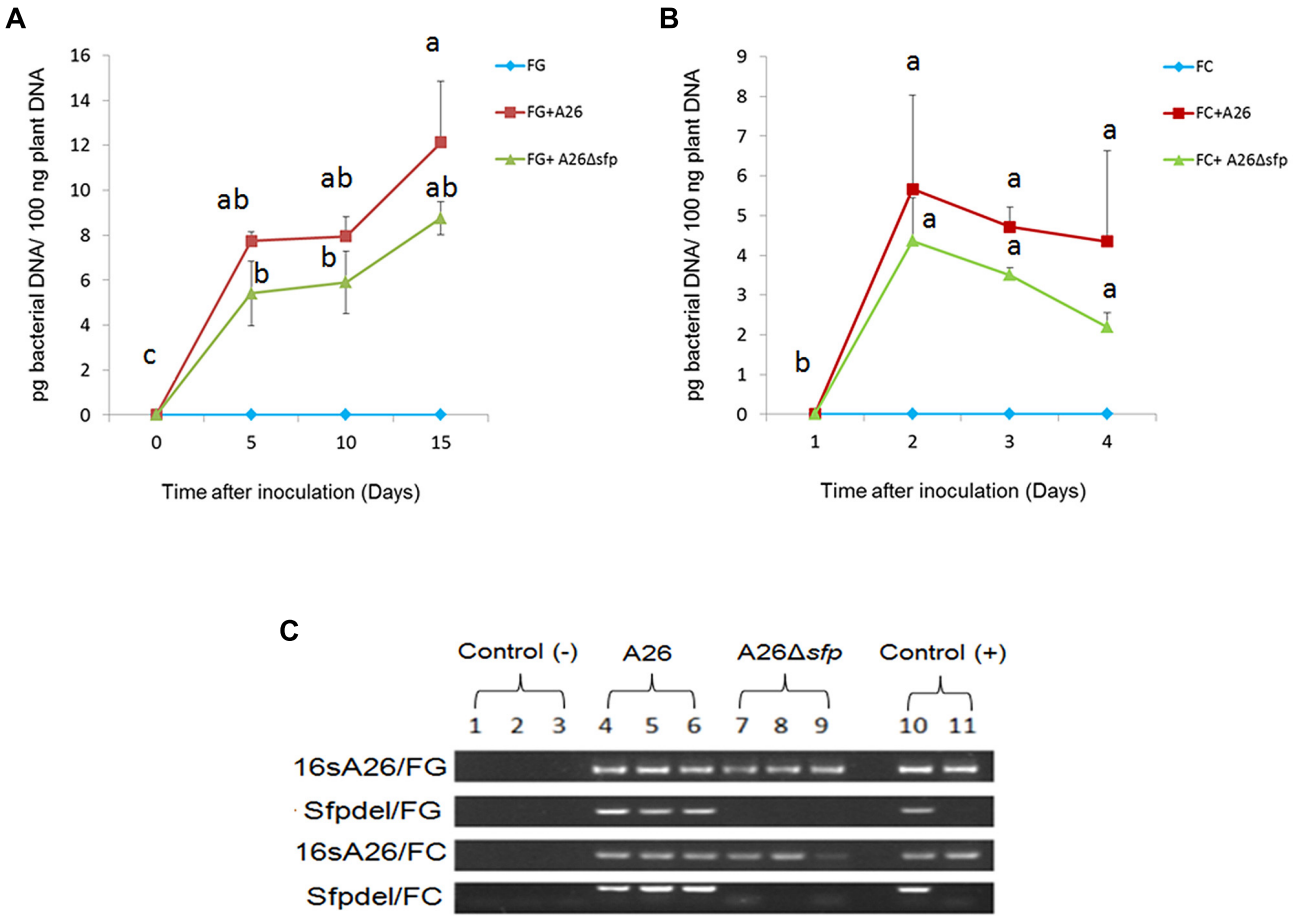
**A26 and A26Δ*sfp* quantification in wheat kernel assay**. QPCR quantification of bacterial DNA extracted from wheat grains inoculated with A26 and A26Δ*sfp* as well as un inoculated wheat grains after 5, 10, and 15 days **(A)** *F. graminearum* and **(B)** *F. culmorum*; (pg bacterial DNA/100 ng plant DNA). Data shown as a means of two experiments; Bars represents SD; Different letters indicate statistically significant differences (*P* ≤ 0.01) based on the LSD test. **(C)** PCR analysis for bacterial DNA using 16S A26 primers (identifying both A26 and A26Δ*sfp*) and sfpdel primers (identifying only A26). DNA extract from pure cultures of A26 and A26Δ*sfp* was used as a positive control while DNA extracted from untreated wheat grains was used as a negative control.

Bacterial DNA in wheat kernel assay was monitored at all three time points using qPCR (**Figure 2**). Increasing the incubation time did not lead to a significant increase in DNA levels after day 5. The only exception was observed on *F. graminearum* treated kernels on day 15 then 12.12 pg bacterial DNA/100 ng plant DNA was detected (**Figure 2**). Using specific PCR primers (**Table 1**) we confirmed the stability of the Sfp-type PPTase mutation at all-time points (**Figure 2B**).

## Discussion

Different strategies have been used to manage FHB (Wegulo, 2012). The challenge with using biocontrol bacteria for this purpose is finding and maintaining effective strains for field applications and monitoring the strains active principle(s) under field conditions. The dual culture plate antagonism assay has often been used as a fast method for screening BCAs *in vitro*. Here we used King’s B medium to compare the antagonistic abilities of a NFS strain E1, SFS strain A26, and A26 Sfp-type PPTase mutant (**Table 2**). The rationale for using A26 strain is that rhizobacteria from harsh environments were previously shown to be effective at enhancing the drought tolerance of host plants (Timmusk et al., 2014a). The same strains were superior in FHB pathogens biocontrol field assays (Timmusk et al., 2014b). Both abilities are likely a consequence of both ACC deaminase activity and the production of broad range of biologically active compounds by the harsh environment isolates (Glick, 2014 and manuscript in preparation). The results presented here show that SFS strain A26 is highly effective in antagonizing *F. graminearum* and *F. culmorum* on the plate assay in comparison to the NFS strain E1 that was tested (**Table 2**). The A26 strain which shows high antagonistic activity on plate assays, was mutagenized to create a deletion its Sfp-type PPTase gene (Timmusk et al., 2015). The A26 Sfp-type PPTase mutant lost its ability to antagonize both pathogens on the plate assay. This result is expected considering that none of the NRPS PKS lipopeptide antibiotics are produced by the mutant strain, and it confirms the role of the compounds in the antagonism (Timmusk et al., 2015). Then the wild-type and mutant strains were studied further in a gnotobiotic system on wheat kernels (**Figure 1**). Compared to plate assays, the system provides a surface for colonization as well as nutrition source that might be used by both the pathogen and the BCA under field conditions. It also allows simultaneous quantitative monitoring of pathogen, BCA A26, and A26Δ*sfp* as well as mycotoxin production. In this system, A26 showed full inhibition of *F. culmorum* and *F. graminearum* by day 5, which did not change during the course of the 15 day studies (**Table 3**; **Figure 1**). The antagonism was followed by qPCR of pathogen, A26 and mycotoxins LC-MS/MS assay which confirm that no pathogens DNA nor mycotoxins were present in the system at any time points (**Figure 2**; **Tables 3** and **4**). The mutant, A26Δ*sfp* lost its ability to antagonize *F. graminearum*, which is the expected result as none of the NRPS PKS lipopeptide antibiotics are produced by the mutant strain (**Figure 1**; **Tables 3** and **4**; Timmusk et al., 2015). Direct antagonism of pathogens is widely considered as the most powerful mechanism employed by soil bacteria against pathogens (Cawoy et al., 2014). *B. subtilis*, the best studied BCA most frequently reported biocontrol mechanisms are connected to non-ribosomally produced cyclic lipopeptides (Ongena and Jacques, 2008; Perez-Garcia et al., 2011; Zeriouh et al., 2011). Lipopeptides which are amphiphilic molecules with an amino or hydroxy-fatty acid integrated into a peptide moiety, interact with the biological membranes of microbial pathogens, including cell leakage and death (Zeriouh et al., 2011). It’s estimated that some *Bacillus* and *Paenibacillus* species devote from 4 to 8% of their genomes to synthesize antibiotics (Cawoy et al., 2014). An examination of the A26 genome indicates that polymyxins, fusaricidins as well as quite a number of potentially new non-ribosomal lipopeptides/antibiotics are mediated by its Sfp-type PPTase. Hence, in the future it is necessary to identify the key regions in the respective synthetase gene clusters and perform knockouts of all known and potential antibiotic candidates (Timmusk et al., 2015). While strain A26Δ*sfp* in a great deal lost its antagonistic ability against *F. graminearum*, the mutant still efficiently antagonized *F. culmorum* (**Figure 1**; **Tables 3** and **4**). On the other hand, a cell free culture supernatant assay showed that the culture filtrates of the mutant were unable to antagonize the pathogen in the kernel assay (**Figure 1**; **Table 4**). What mechanism could explain the different effect of the mutant strain in the kernel assay? The Sfp-type PPTase activates peptidyl carrier protein domains (Quadri et al., 1998; Beld et al., 2014; Bunet et al., 2014). During the compound assembly, the biosynthesis intermediates are attached to carrier protein domains of NRPS and PKS via a phosphopantetheinyl arm. The post-translational modification of the domains with 4′-phosphopantetheyinyl as catalyzed by Sfp-type PPTase is crucial for the activation of NRPS and PKS (Beld et al., 2014; Bunet et al., 2014). The mutant A26Δ*sfp*, which is incapable of producing the enzymatically active 4′ phosphopantetheinyl transferase, in turn results in a *P. polymyxa* A26 mutant strain lacking enzymatically active NRPS and PKS and lipopeptide production (Timmusk et al., 2015). Using specific PCR primers (**Table 1**) we regularly confirmed the stability of the mutant and wild-type strain, (**Figure 2C**). These results indicate that fusaricidins, generally believed to be the mechanism of *P. polymyxa* action against *Fusarium* sp. may not be the only mechanism functioning on the A26 in biocontrol of *F. culmorum*. A26Δ*sfp* culture filtrates failed to antagonize *F. culmorum* in kernel assay in contrast to A26Δ*sfp* cells treatment then we detected the antagonism against the pathogen (**Figure 1**). This indicates biofilm involvement in the antagonism and is in accordance with our former findings showing that niche exclusion, i.e., antagonist biofilm occupation of the pathogen colonization sites, is responsible for biocontrol (Timmusk et al., 2003, 2009b; Haggag and Timmusk, 2008). Microbial biofilms are comprised of cells and extracellular matrix and can produce a protective layer around infection sites. The dense biofilm matrix limits diffusion of compounds secreted by bacteria and these are therefore concentrated at pathogen infection sites of action. This knowledge is important to be taken into consideration for further selection of biocontrol agents in field conditions where the *F. culmorum* content is estimated to be high. In parallel with selection of the highest lipopeptide producers, the isolate’s biofilm production should be taken into consideration.

While A26 Sfp-type PPTase mutant fully antagonized *F. culmorum* and *F. graminearum* growth was also decreased in the kernel assay first two time points (**Table 3**). At the same time even if only low levels of the pathogens were detected, significant amounts of both mycotoxins were produced by the pathogen at the times points (**Table 4**). This confirms the importance of parallel monitoring of pathogen growth and mycotoxins produced.

In the work reported here we established a progressive screening method for FHB BCAs: plate assays combined with a wheat kernel assay. The system allows robust screening of high number of *P. polymyxa* isolates from harsh environments (Timmusk et al., 2011, 2014a). In the A26 case, plate assays with wild type and its Sfp-type PPTase mutant confirm that NRPS/PKS products have critical importance for the strain antagonistic ability (**Table 2**). Studies with the more complex system employing wheat kernels show, however, that in case of *F. culmorum* the BCA-enhanced biofilm formation may be of major importance in antagonizing the pathogen. Simultaneous qPCR monitoring of A26 and pathogens combined with mycotoxin assays supports this finding (Figure 2 2; **Tables 3** and **4**). The system will be used now to monitor the efficiency of A26 formulation strategies. Moreover, our results confirm that external complementation with A26 metabolite extracts efficiently restores its wild type biofilm formation level indicting the Sfp-type PPTase mediated NRPS/PKS compounds direct involvement in A26 biofilm formation (Timmusk et al., 2015). Dual culture plate and kernel assays with additional knockout mutants of single NRPS/PKS compounds should reveal which of the lipopeptides are active in the observed biofilm formation and antagonistic activity. The qPCR monitoring optimized for the A26 can now be used with the formulated agent monitoring on field application. Furthermore, in parallel with the BCA monitoring the system for the lipopeptide monitoring proven to be involved in A26 biofilm formation and antagonism will be designed. To maintain the BCA active principles during storage is one of the challenges with biocontrol strains. Therefore it is quite important to have a simple system for routine screening of stored A26 and further FHB active BCAs.

It is clear that more studies need to be performed for strain A26 prior to field application. However, the results obtained so far show the importance of combining the strategies described here in order to develop efficient FHB biocontrol agents for field application.

## Conflict of Interest Statement

The authors declare that the research was conducted in the absence of any commercial or financial relationships that could be construed as a potential conflict of interest.
